# Preparation of thick silica coatings on carbon fibers with fine-structured silica nanotubes induced by a self-assembly process

**DOI:** 10.3762/bjnano.8.116

**Published:** 2017-05-26

**Authors:** Benjamin Baumgärtner, Hendrik Möller, Thomas Neumann, Dirk Volkmer

**Affiliations:** 1 Chair of Solid State and Materials Chemistry, University of Augsburg, 86159 Augsburg, Germany; 2 Schwenk Zement KG, Ulm 89077, Germany; 3 Schwenk Zement KG, 97753 Karlstadt, Germany

**Keywords:** biomimetic silicification, carbon fiber, self-assembly, silica nanotubes, sol–gel process

## Abstract

A facile method to coat carbon fibers with a silica shell is presented in this work. By immobilizing linear polyamines on the carbon fiber surface, the high catalytic activity of polyamines in the sol–gel-processing of silica precursors is used to deposit a silica coating directly on the fiber’s surface. The surface localization of the catalyst is achieved either by attaching short-chain polyamines (e.g., tetraethylenepentamine) via covalent bonds to the carbon fiber surface or by depositing long-chain polyamines (e.g., linear poly(ethylenimine)) on the carbon fiber by weak non-covalent bonding. The long-chain polyamine self-assembles onto the carbon fiber substrate in the form of nanoscopic crystallites, which serve as a template for the subsequent silica deposition. The silicification at close to neutral pH is spatially restricted to the localized polyamine and consequently to the fiber surface. In case of the linear poly(ethylenimine), silica shells of several micrometers in thickness can be obtained and their morphology is easily controlled by a considerable number of synthesis parameters. A unique feature is the hierarchical biomimetic structure of the silica coating which surrounds the embedded carbon fiber by fibrillar and interconnected silica fine-structures. The high surface area of the nanostructured composite fiber may be exploited for catalytic applications and adsorption purposes.

## Introduction

Carbon fibers are widely used as reinforcement in ceramic, metal matrix and carbon composites because of their outstanding properties, such as high specific strength, a high Young’s modulus, low expansion coefficient and relative flexibility [1]. For the application in adsorption processes, carbon fiber felts and carbon cloths can substitute activated carbon in its traditional powdered or granular forms. For this application, the carbon fiber material has to be activated by means of physical (i.e., thermal) or chemical activation processes. The contiguous structure of activated carbon cloths bears several advantages in comparison to the powdered carbon materials, such as suitability for electrical and electrochemical procedures. In addition, activated carbon cloths are light materials that can be arranged in different configurations. The same features that promote the application in the field of adsorption enable the adaption to catalytic applications [2]. For example, cobalt diselenide nanoparticles can be grafted onto a carbon fiber felt creating a three-dimensional hydrogen evolution cathode based on the high conductivity of carbon fibers [3].

A primary objective of the present study is the deposition of nanostructured silica onto the surface of carbon fibers. Among other techniques of covering the carbon fiber surface with a silicon dioxide layer (or other ceramic coatings), the most common ones are chemical vapor deposition (CVD) [4], liquid phase impregnation [5] and sol–gel techniques [6]. Even though the CVD method leads typically to a uniform and dense surface coating, low deposition rates can make this process time-consuming and expensive. The sol–gel method is advantageous owing to the low processing temperature, inexpensive and commercially available precursors and the possibility to control the ceramic composition. For instance, modification and variation of the alkyl and alkoxy groups of the employed silica precursors allow for controlling the composition of anti-oxidative SiC/SiO_2_ coatings [6]. The sol–gel method for coating carbon fibers with silicon dioxide (or other ceramic coatings) typically utilizes metal alkoxides, e.g., tetramethyl orthosilicate, which are hydrolyzed in water/ethanol mixtures containing hydrochloric acid to obtain a sol. The coating procedure of carbon fibers involves three steps: dipping the carbon fiber into the sol solution, a drying process at temperatures below 100 °C and high-temperature treatment at several hundred degrees centigrade [1,6].

The most important reason to develop these methods, which are reported in literature with the objective of coating carbon fibers with silicon dioxide and ceramic layers, is oxidation protection of carbon fibers at elevated temperatures. The good mechanical properties of carbon fibers are preserved under vacuum and inert atmospheres up to temperatures higher than 2000 °C. However, in an oxidative environment, carbon fibers show a low oxidation resistance, i.e., even at temperatures as low as 400 °C, an oxidation can be observed in air [6]. This limitation can be overcome by protective coatings of carbon fibers. Especially the application of ceramic compound coatings is an established method for improving the fiber’s oxidation resistance [1]. Ceramic coatings, such as carbide [1,7–8], nitride [9] and oxide [10] were proven to be a successful way to retain the mechanical performance of carbon fibers in oxidative atmospheres at higher temperatures.

Acid or base catalysis is necessary for the hydrolysis and the polycondensation of silica precursors to obtain a sol. In addition, amines and polyamines can facilitate the silicic acid polycondensation [11–13]. A biomimetic system, which employs linear poly(ethylenimine) (LPEI) and was introduced recently by Jin et al., is capable of both catalyzing silicification and directing the silica morphology [14–16]. LPEI exhibits a structurally uniform, linear backbone, which contains secondary amine groups. In contrast to hyper-branched poly(ethylenimine), which contains both secondary and tertiary amine groups, LPEI can form several crystalline (pseudo-) polymorphic phases, which leads to its characteristic low water-solubility at ambient temperature. If an aqueous LPEI suspension is heated up to 80 °C, it forms a transparent solution and upon cooling down to room temperature, the polymer recrystallizes as a dihydrate in the form of linear aggregates [14]. If silica precursors are used, the formed aggregates can be used as catalytically active templates for silicon dioxide formation. In contrast to branched poly(ethylenimine), the LPEI molecules are insoluble in water, which confines the silica deposition onto the surface of the LPEI aggregates. That way, it is possible to create silica-polyamine hybrid particles, composed of bundled silica nanotubes and nanoribbons [17–18]. The diameter of these silica building units is restricted by the nature of LPEI, ranging from 30 to 150 nm [16]. It was deduced from DSC and XRD measurements that the linear LPEI aggregates consist of a crystalline core, which functions as a template, and an amorphous shell, i.e., free ethylenimine units, which direct the silica deposition to the aggregates’ surface. As soon as the amorphous shell is covered by silica, no further significant deposition takes place [16].

A considerable number of factors can influence the shape of the primary LPEI aggregates and, consequently, the morphology of the mineralized silica particles. This can be realized by varying the LPEI and/or silica precursor concentration, timespan of silicification [19], media modulation (i.e., partial replacement of water by methanol or other solvents) [20], pH value [21], or by introducing acidic and basic additives that can interact with LPEI [22] and other proton donors [23]. Further modification of the silica morphology can be achieved by adding metal ions that form complexes with the secondary amine groups of LPEI [24–26]. The coordination interactions between the polyamine and the metal ions do not block the LPEI crystal growth, but largely change the crystalline morphology [27].

A prominent feature of this polyamine mediated biomimetic silicification process is the capability of LPEI to undergo self-assembly on arbitrary substrates. A sufficient molecular-level interaction of LPEI with the substrate is essential to create a continuous coating with vertically aligned linear aggregates, which can be mineralized to produce a silica “nanograss” film on the substrate. For example, a nanograss film can be prepared on a glass surface, but not on a hydrophobic polystyrene substrate. Treatment of polystyrene with sulfuric acid introduces sulfonate groups, and thus the possibility for hydrogen-bonding between –NH and –SO_3_H. The modified polystyrene surface is suitable for the nanograss formation demonstrating the importance of sufficient molecular-level interactions between substrate and polyamine [28]. The nanograss films on glass were shown to have a potential application for surface wettability design [29–31].

In our study we focused on the coating of chopped carbon fibers and carbon fiber felts with silica by polyamine mediation. The polyamine is immobilized at the carbon fiber surface either by a covalent linkage or by a self-assembly process of linear polyamines, i.e., LPEI. The covalent linkage of short-chain amines to the carbon fiber surface has already been reported in literature and it was shown that a high surface amine concentration of carbon fibers can enhance adhesion to both polyurethane and epoxy resins matrices [32]. A chemical modification of carbon fibers with tetraethylenepentamine (TEPA) was presented as a possible option. For an effective binding of TEPA via amide functional groups, the number of carboxylic acid groups on the carbon fiber surface can be increased by nitric acid oxidation [33]. The catalytic activity of the polyamines, which are localized on the carbon fiber’s surface, is exploited by depositing silica site-selective onto the carbon fiber at close to neutral pH conditions. In comparison to conventional coating procedures, which are typically limited to dimensions in the nanometer range, the suggested process of LPEI mediated immobilization of silica nanotubes onto carbon fibers allows covering the surface with an amorphous silica shell that can reach an extent of several micrometers in thickness. In addition to the new approach of polyamine mediated silica deposition onto carbon fibers, the presented method by means of LPEI immobilization is able to introduce a silica shell with a nanostructured surface. This preparation route gives access to a new hybrid material that combines the properties of both the carbon fiber substrate and the nanostructured silica.

For future applications, the combination of both materials might extend the range of application of carbon fibers for adsorption purposes and catalytic issues. Properties of carbon fibers, such as high flexibility or low heat and mass transfer resistance, could be advantageous compared with the traditional powder or pellet form of silica based catalysts. For example, Joule heating of the contiguous carbon fiber felts offers additional benefits regarding desorption for their in situ regeneration in volatile organic compounds treatment processes [2]. Moreover, silica surfaces are suitable for adsorption of electron deficient organic molecules enabling an application of silica coated carbon fibers for adsorption issues and waste water treatment [34]. For catalytic applications, the high surface area of the silica shell could be used either as a catalyst support or as a catalytic active material. The second possibility can be fulfilled for instance by introducing a titania precursor in the mineralization process. A silica/titania mixture is able to catalyze liquid phase epoxidation [35], the NO_x_ abatement under UV irradiation [36] or the photocatalytic decomposition of low-surface-energy organic components attached to the nanostructured surface [19].

As will be presented in this work, LPEI shows the ability for complexation of metal ions, e.g., copper, not only leading to a change in the morphology of the silica shell, but also incorporating metal centers into the organic template core. A calcination process removes the LPEI template and provides access to the metal centers located at the inner wall of the silica nanotubes enabling potential catalytic reactions within the pores of the silica shell. A further conceivable application can be evaluated with a reference to the oxidation protection due to the possibility of sintering the fine-structure at elevated temperatures associated with a carbothermal reduction to silicon carbide [6].

## Results and Discussion

### Deposition of thin silica films onto carbon fiber surfaces via chemical modification with tetraethylenepentamine

Besides embedding carbon fibers in a nanostructured silica shell, the new approach presented in this work is the localization of polyamines as a catalyst for silica precursor hydrolysis and condensation on the carbon fiber surface. The following polycondensation can be accomplished at neutral pH due to the polyamine catalyst which is immobilized on the fiber surface. In addition, secondary silica particle formation in the dispersing phase can be minimized due to the absence of any catalyst in the continuous phase. As a reference, a direct silica deposition onto a carbon fiber by means of an acid catalyzed sol–gel process was tested by employing sodium silicate solution at pH 2 (Supporting Information File 1, Figures S1 and S2). As can be seen from these reference experiments, nucleation takes also place in the continuous phase, i.e., the fiber dispersing phase, leading to the formation of separate (non-immobilized) silica particles.

**Figure 1:**
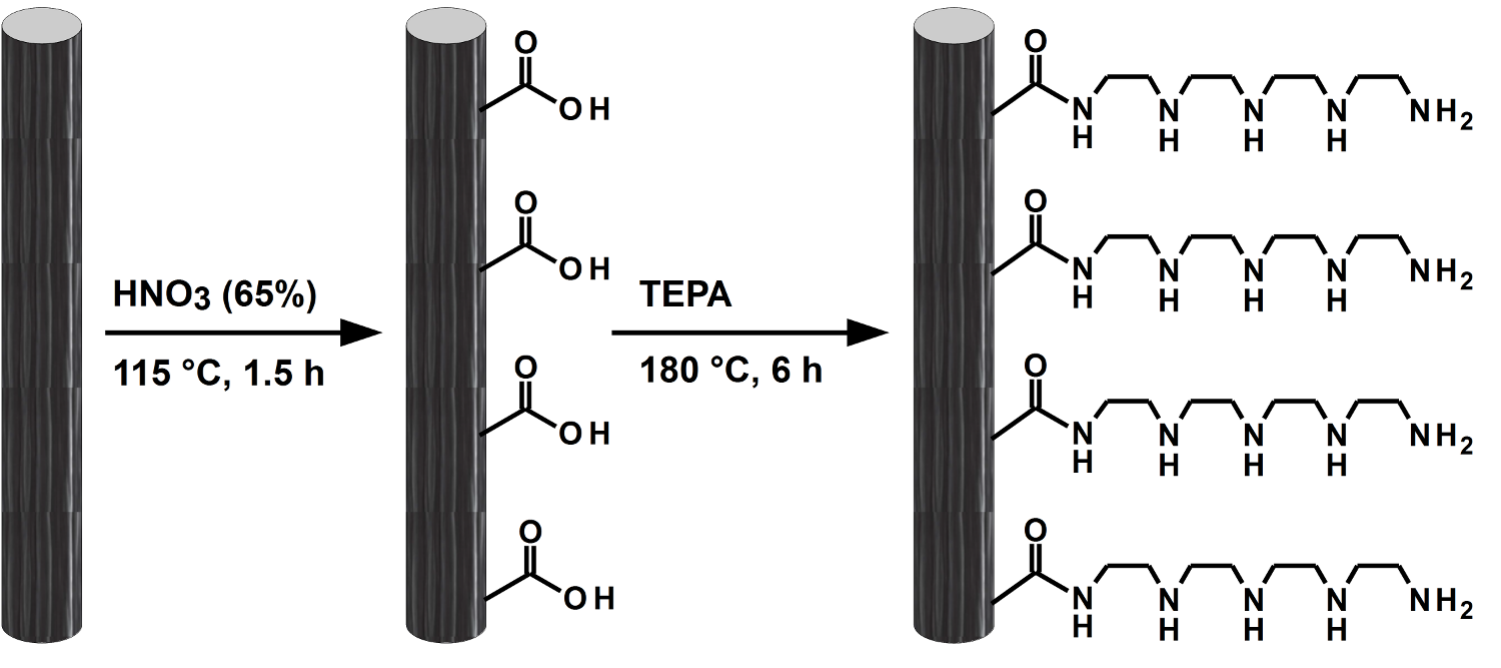
Schematic illustration of the covalent linkage of tetraethylenepentamine to carbon fibers via wet-chemical oxidation and a subsequent amide formation.

In principle, catalytic active polyamines can be localized on the surface of carbon fibers by covalent linkage or by weak van der Waals forces. The latter will be demonstrated for the self-assembly of long-chain polyamines, namely LPEI, in the following chapter. Tetraethylenepentamine was chosen for covalent linkage to the fiber surface (Figure 1). The number of surface-exposed carboxylic acid groups was increased by wet-chemical oxidation with 65% nitric acid at 115 °C over a period of 90 min. Grafting the short-chain polyamine via amide functional groups was achieved by heating the previously oxidized carbon fibers at 180 °C in an excess of TEPA for 6 h. The possibility of a successful grafting by heating the fibers in an excess of TEPA was already demonstrated elsewhere [32]. In the present case, the presence of the polyamine at the carbon fiber surface was confirmed by XPS measurements (relative amounts: C 88.0%; N 4.0%; O 8.0% for oxidized fibers without TEPA grafting; C 75.5%; N 16.0%; O 8.5% for fibers with TEPA grafting) and quantified by a mass loss of 4.2% via thermogravimetric analysis by heating in nitrogen atmosphere. Silica deposition onto the as prepared fibers was conducted with 30 vol % tetramethyl orthosilicate (TMOS) in a mixture of water and ethanol (one-to-one by volume) within 40 min.

**Figure 2:**
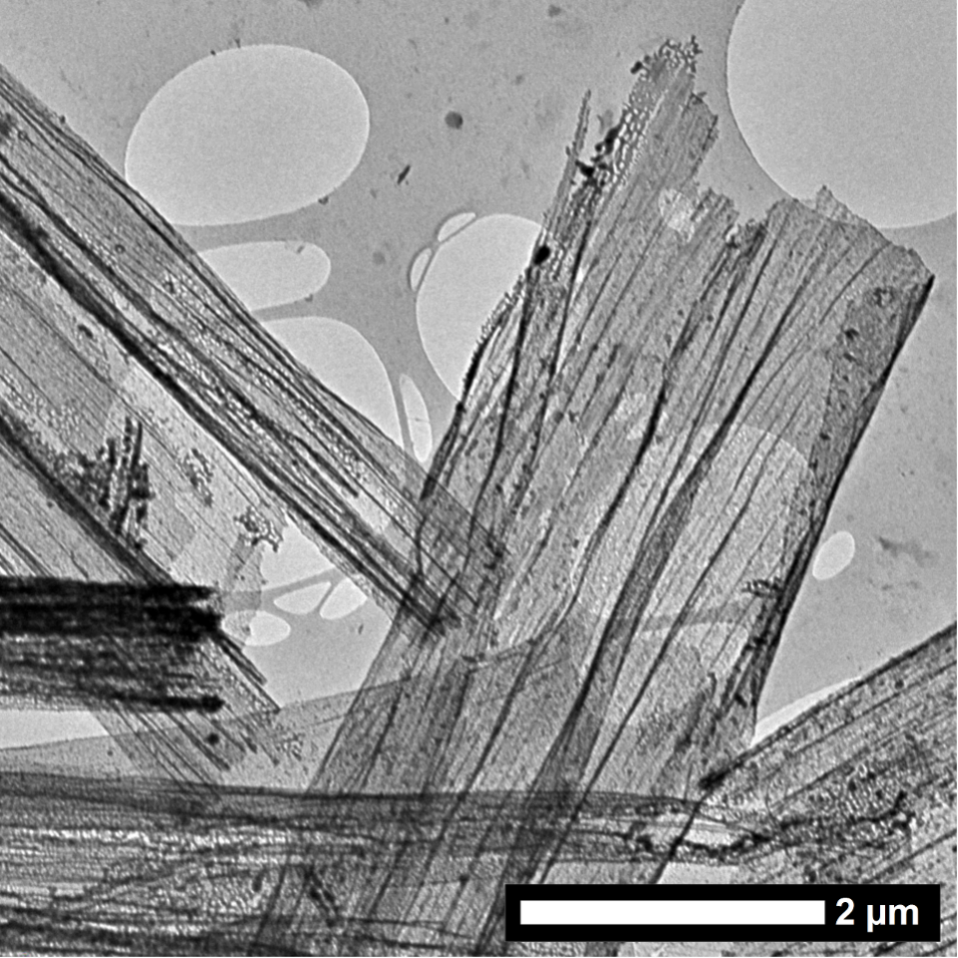
Transmission electron micrograph of thin silica shells remaining after calcination of the TEPA-modified carbon fibers.

The presence of the polyamine catalysts facilitates the silica deposition, and furthermore, the localization of the silica on the carbon fiber surface. The presence of silica on the fiber surface was confirmed by EDX analysis and quantified to 0.9 wt % of the total composite weight by means of thermogravimetric analysis. Transmission (Figure 2) and scanning electron microscopic observations (Supporting Information File 1, Figure S3) of the fluffy residue after the thermogravimetric measurement elucidate a thin but coherent nature of the silica film which is left after calcination of the carbon fiber in air atmosphere.

**Figure 3:**
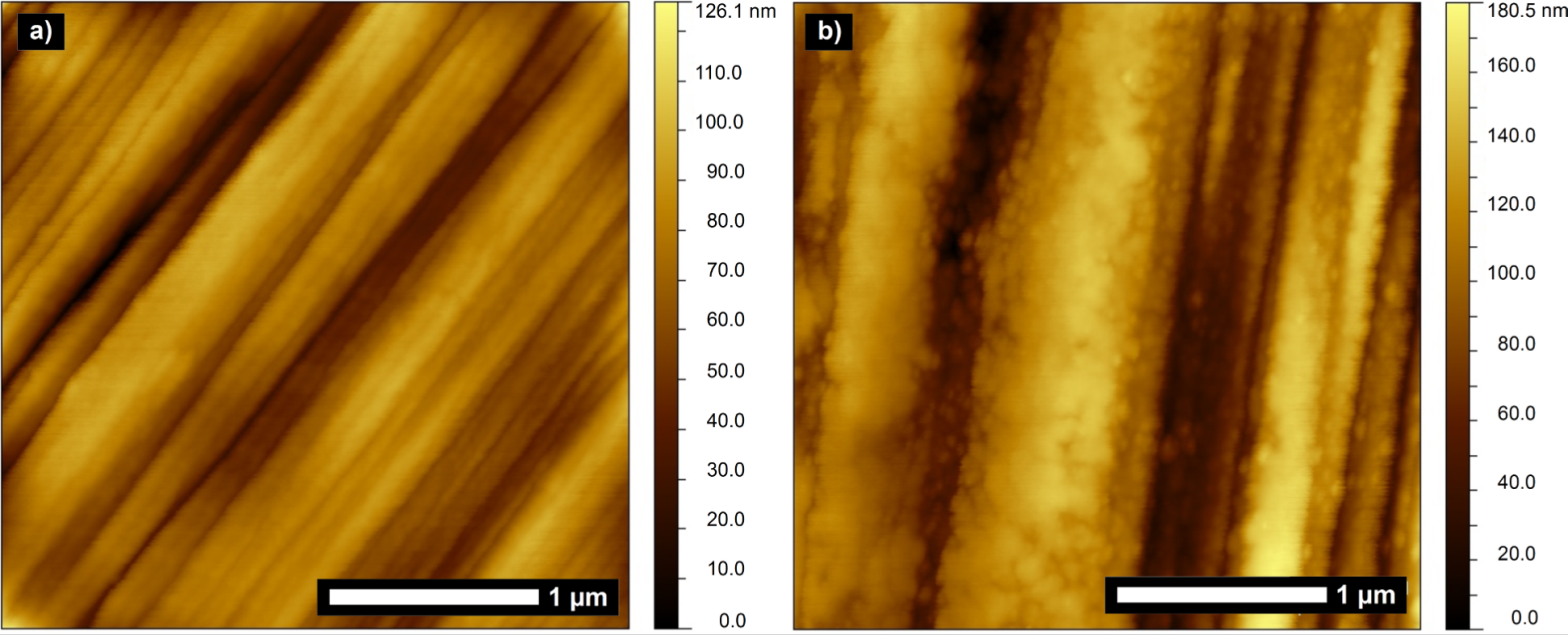
Atomic force micrographs of carbon fibers after 40 min in 30 vol % TMOS solutions with a pretreatment of a) nitric acid oxidation and b) nitric acid oxidation and subsequent TEPA grafting.

For comparison, atomic force microscopy was performed on nitric acid oxidized fibers with and without TEPA grafting. Both kinds of fibers were treated in the same way for 40 min in a 30 vol % containing TMOS solutions. As can be seen from the AFM scans of these carbon fibers treated with silica precursor solution, no silica deposition occurs for the polyamine-free system whereas the TEPA modified carbon fiber surface is densely coated with silica (Figure 3). The absence of silica in the polyamine-free system can be further confirmed by means of EDX and TGA analysis revealing no evidence for silica being present on the fiber surface. This proves the importance and necessity of polyamine presence on the fiber surface for the proposed wet-chemical silica coating method.

Due to the high catalytic activity of the polyamine with regards to the silica polycondensation, a diminished TMOS concentration of as low as 3 vol % is sufficient for silicification. Increasing the reaction time beyond 40 min up to 24 h does not increase the amount of silica deposited on the fiber surface, as can be seen from TGA. Hence the deposition route via TEPA modified fibers makes only thin silica coatings in the nanometer range possible, while preventing the formation of separate silica particles in the dispersing phase.

### Silicification of carbon fibers with surface-confined LPEI aggregates

While the short-chain polyamine TEPA was covalently bound to the carbon fiber surface, the suitability of linear poly(ethylenimine) for a self-assembly process can be used to localize the polyamine on the fiber surface via van der Waals forces. This way, a coating of the fiber with LPEI aggregates can be obtained which serves as both catalyst and template in the following mineralization step. The nature of LPEI enables access to a nanostructured patterning of the resulting silica shell.

### LPEI/silica composite particles and their immobilization on carbon fibers

**Figure 4:**
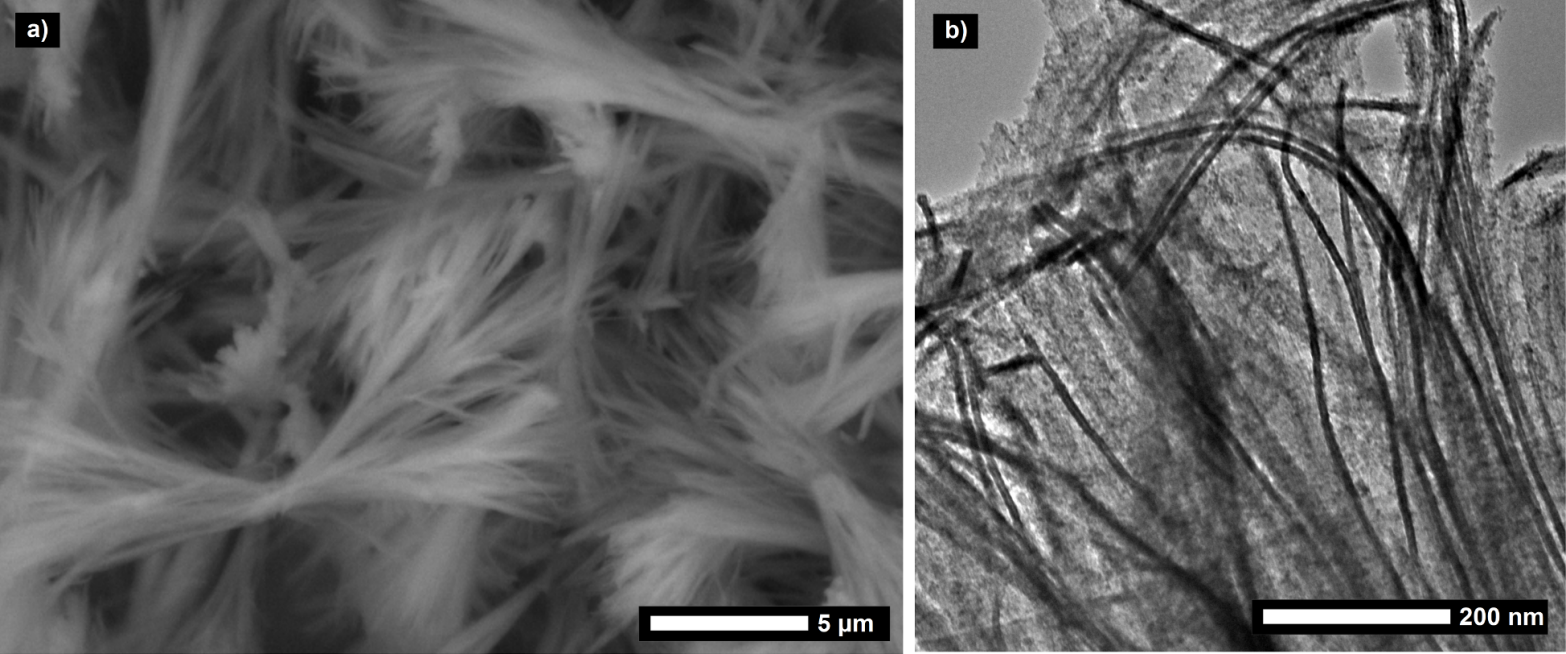
a) Scanning and b) transmission electron micrograph of mineralized LPEI aggregates.

For generating LPEI aggregates, which can serve as a template for silica deposition, typically 1 wt % of polymer was suspended in water and heated to about 80 °C. Upon cooling down the formed transparent solution to room temperature, LPEI recrystallizes in a form of fibrous bundles, which can be highly branched. The fiber bundle morphology is preserved in the subsequent mineralization process initialized by addition of 3 vol % silica precursor tetramethyl orthosilicate (TMOS) in a mixture of ethanol and water (1:1, v/v). The polymer aggregates are encapsulated by a silica shell in a very short timeframe of 40 min. It has to be emphasized that no addition of any further acid or base catalyst is necessary to induce the observed rapid hydrolytic condensation of TMOS. This can be explained by the high density of catalytically active ethylenimine brushes enriched on the surface of the aggregates. Electron microscopy images visualize the fibrous morphology of the mineralized silica bundles (Figure 4). The silica content of the hybrid particles is about 70 wt %, as determined by thermogravimetric analysis in air atmosphere (Supporting Information File 1, Figure S4), also proving that the particles are hybrid particles of organic LPEI and inorganic silica. It was already reported in literature that a calcination process creates nanotubes by removal of the LPEI core while the overall fibrillar structure is preserved [16]. Isolated single nanotubes have a uniform diameter of about 10 nm and an inner accessible hollow of 3 nm [17–18].

**Figure 5:**
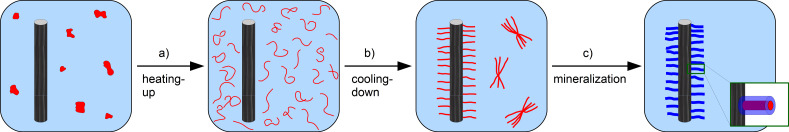
Schematic illustration of LPEI mediated silicification of carbon fibers: a) chopped carbon fibers and LPEI are suspended in water and heated to a temperature above 80 °C resulting in a molecular-dispersed solution of LPEI; b) cooling-down of the solution to room temperature causes LPEI to recrystallize in the form of both separate LPEI aggregates and on the fiber surface self-assembled LPEI aggregates; c) the LPEI coated carbon fibers are separated and mineralized in a solution of TMOS in an ethanol/water mixture leading to silica shells which are surrounding the LPEI aggregates.

Chopped carbon fibers were pretreated by calcination to remove sizing agents and to evaluate the immobilization suitability of linear poly(ethylenimine) for the fiber surface. To enable a self-assembly of the LPEI molecules on the carbon fiber’s surface, the chopped carbon fibers were introduced to the same LPEI suspension as described above for the preparation of the LPEI/silica hybrid particles. As long as the molecular-level interactions between the polyamine and the fiber surface are sufficient, the LPEI self-assembles on the substrate after cooling down the transparent polymer solution (Figure 5).

**Figure 6:**
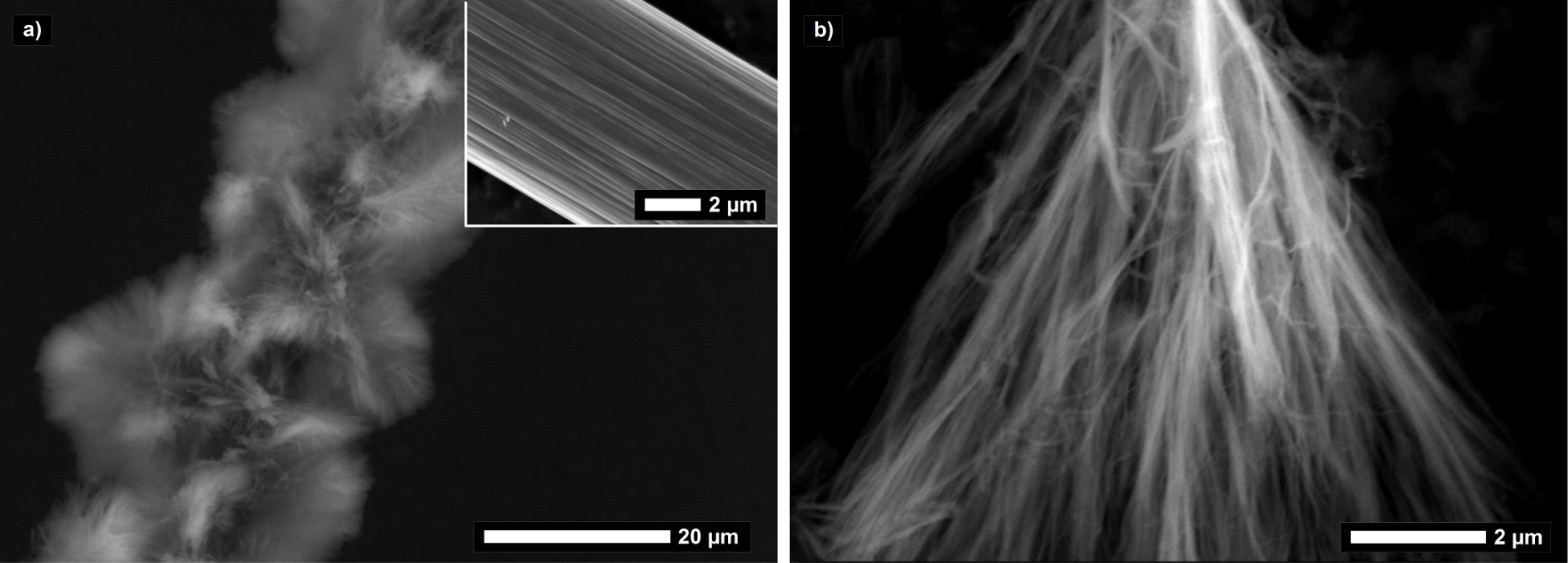
Scanning electron micrographs of a) a carbon fiber coated with a silica shell mediated by LPEI (inset: surface patterning of the neat carbon fiber) and b) close-up image of a silica shell fragment.

The LPEI aggregates are relatively stable due to their crystalline nature so that it is possible to separate the non-immobilized LPEI aggregates from the LPEI covered carbon fibers prior to silicification without provoking any damage of the morphology of the polyamine aggregates. This can be realized by means of a coarse-meshed sieve in order that the LPEI which is not immobilized on carbon fibers can be regained. Scanning electron microscopic observations of the mineralized fibers show that the fibers are densely covered with linear silica structures (Figure 6). The diameter increases from 7 µm, for the neat fibers, to about 25 µm, for the composite fibers including the nanostructured silica shell. To estimate the composition of the hybrid fiber, a thermogravimetric analysis was performed both in nitrogen and in air (Supporting Information File 1, Figure S5). The LPEI decomposes completely in non-oxidative nitrogen atmosphere. A weight loss of about 18 wt % of the total weight is caused by LPEI and water. The same sample was then heated in air atmosphere to detect a weight loss of about 34 wt % caused by decomposition of the carbon fibers. Consequently, the residue of about 48 wt % of the total weight is the amount of silica in the hybrid fiber. The composition of the sheer silica shell, neglecting the carbon fiber, is with 73 wt % silica almost corresponding to the silica content in the separate LPEI mediated silica particles with a value of 70 wt %.

### Control over the silica shell morphology and incorporation of metal ions

**Figure 7:**
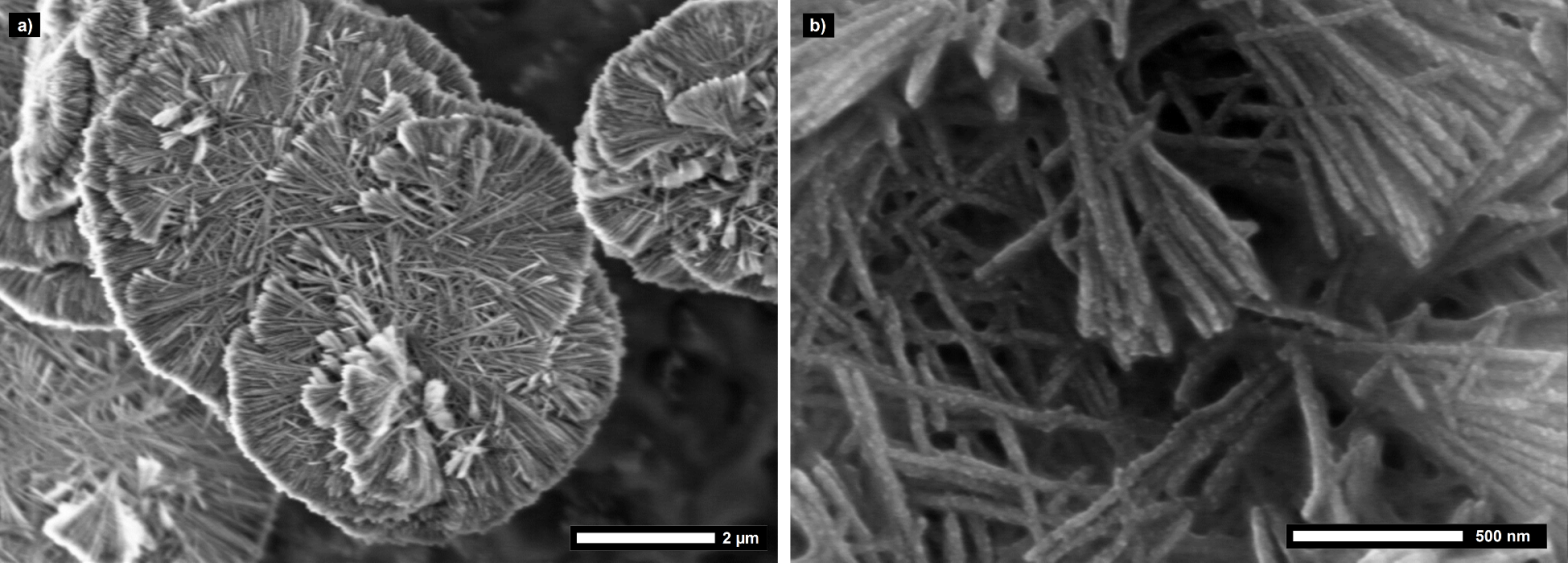
Scanning electron micrographs of mineralized LPEI aggregates modified by copper nitrate addition, a) disk-like morphology of a complete particle, b) close-up image of the linear nanostructures.

**Figure 8:**
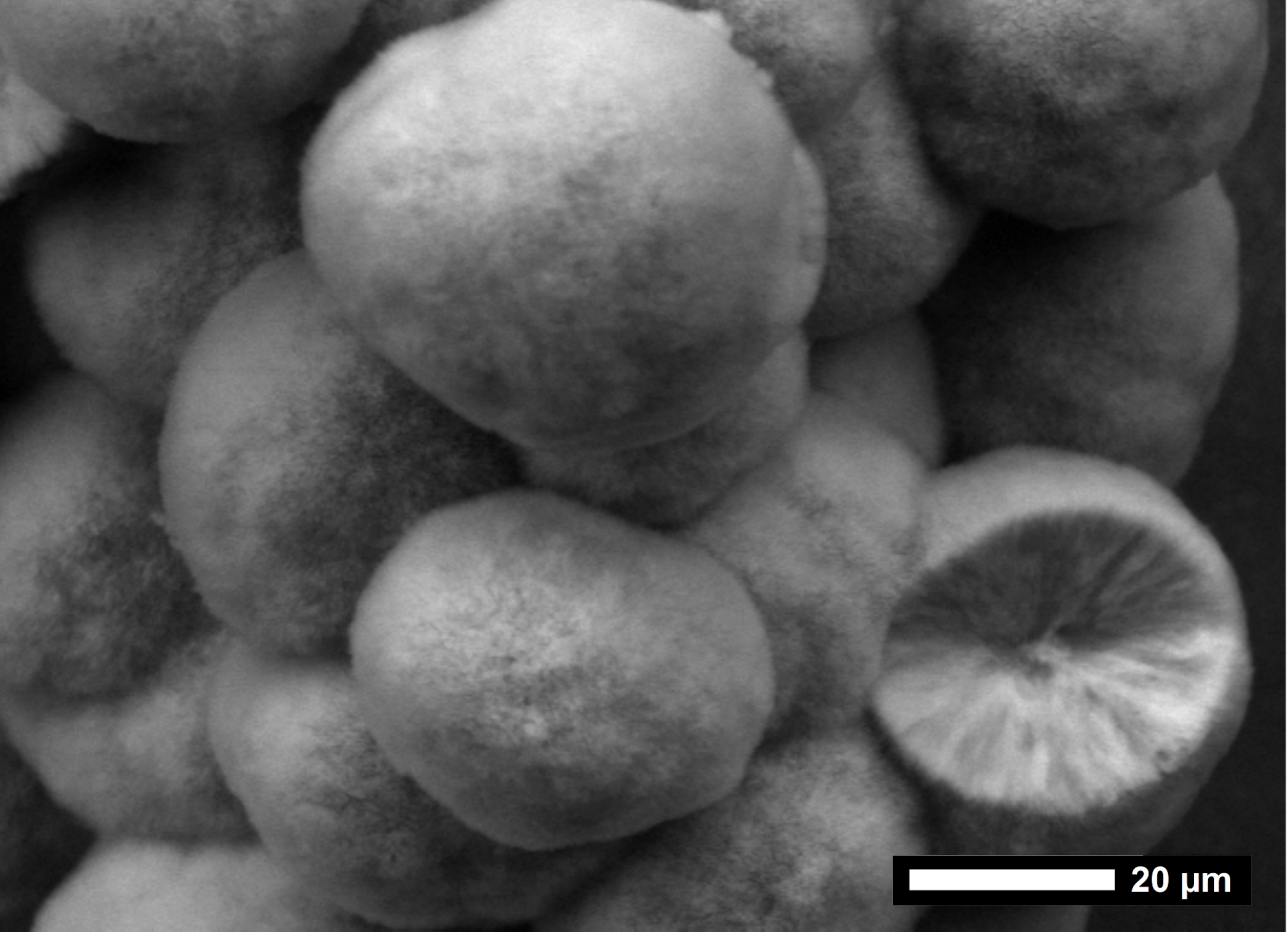
Scanning electron micrograph of mineralized LPEI aggregates modified by calcium nitrate addition.

A main advantage of the LPEI mediated coating process is the facile control over the silica shell morphology. Multiple synthesis parameters can be exploited to control the nature of the silica coating. A more rapid cooling of the carbon fiber suspension leads to smaller LPEI aggregates and consequently a thinner silica shell. For example, a composite fiber diameter of only 10 µm instead of 25 µm is obtained for cooling in an ice-water bath over 5 min (Supporting Information File 1, Figure S6). Additionally, a significant influence on the morphology can be achieved by addition of metal ions. Owing to complex formation between the polyamine and the metal ions, the hierarchical structure is modified by a metal salt addition. The ratio between metal ion and ethylenimine monomer unit was set to 1:30. The hybrid silica particles prepared by addition of copper(II) ions exhibit for this ratio a pronounced two-dimensional and disk-like morphology with a diameter of a single silica fiber of about 30 nm (Figure 7). Usage of calcium(II) ions alters the morphology to spherical structures (Figure 8). Calcination of the metal containing silica particles at 475 °C for 90 min leads to a removal of the LPEI core resulting in hollow silica nanotubes which include metal centers in their inner pores. The BET surface area increases for the copper incorporated silica particles from 140 m^2^/g to 480 m^2^/g, as determined by nitrogen gas sorption measurements.

**Figure 9:**
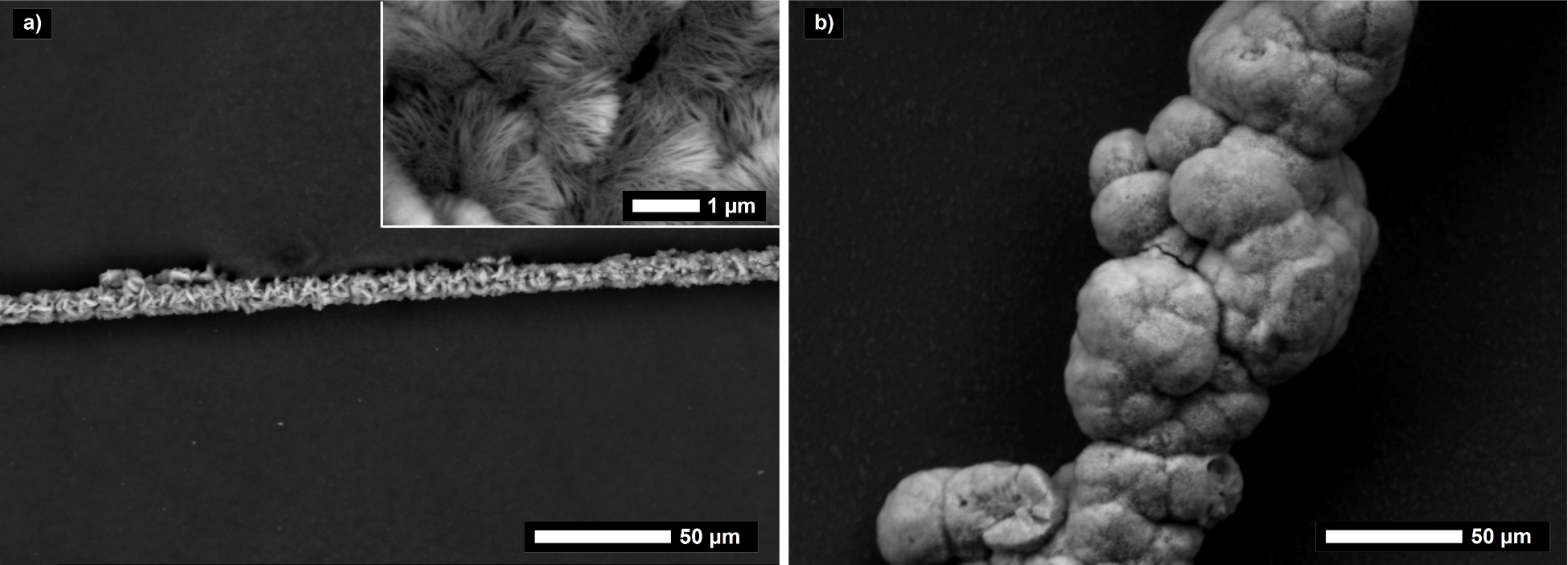
Scanning electron micrographs of carbon fibers coated with a silica shell mediated by LPEI with addition of a) copper(II) and b) calcium(II) nitrate (inset: fine-structure for copper addition ).

The overall morphology is more or less preserved for the immobilization of these modified aggregates on the carbon fiber surface. Addition of copper(II) ions leads to two-dimensional, fan like structures located on the fiber surface, whereas calcium(II) ions redirect the morphology towards a pronounced three-dimensional structure (Figure 9). The diameter of the composite fiber reaches 12 µm in the case of copper(II) ions and up to 100 µm for the incorporation of calcium(II) ions accompanied with a less smooth coating. Thus metal ions can be incorporated in the silica shell concomitant with an influence on the morphology of the fine-structure and the overall silica shell thickness.

The nanostructured silica shell of LPEI mediated silicified carbon fibers is relatively robust. The fine-structure can withstand for example prolonged sonication in an ultrasonic bath and a calcination process at a temperature of 800 °C. However, the fine-structure will be sintered by thermal annealing at higher temperatures, what restricts the usability for catalytic applications at higher temperatures. For instance, this can be observed for isothermal treatment at 1100 °C for 15 h in an argon atmosphere associated with disappearance and coalescence of the fine-structure (Supporting Information File 1, Figure S7).

### Variation of the fiber substrate

The surface of carbon fibers of which the sizing agent is removed by calcination at 400 °C is attractive enough for the molecular-level interactions with the polyamine. Several other modifications of carbon fiber surfaces were examined confirming that an increase in hydrophilicity is beneficial for the LPEI self-assembly process. For example, a dense coating can be realized for anodic oxidized fibers, sulfonated fibers (obtained by treatment with sulfuric acid, Supporting Information File 1, Figure S8) and wet chemical oxidized fibers. It has to be stated that the nature of the surface can influence the resulting morphology. In comparison to the surface of calcined fibers, the sulfonated fiber surface is (with almost the same shell thickness) more dense coated with silica fine-structures leading to a higher silica amount of 56 wt % of the total weight of the composite fiber.

**Figure 10:**
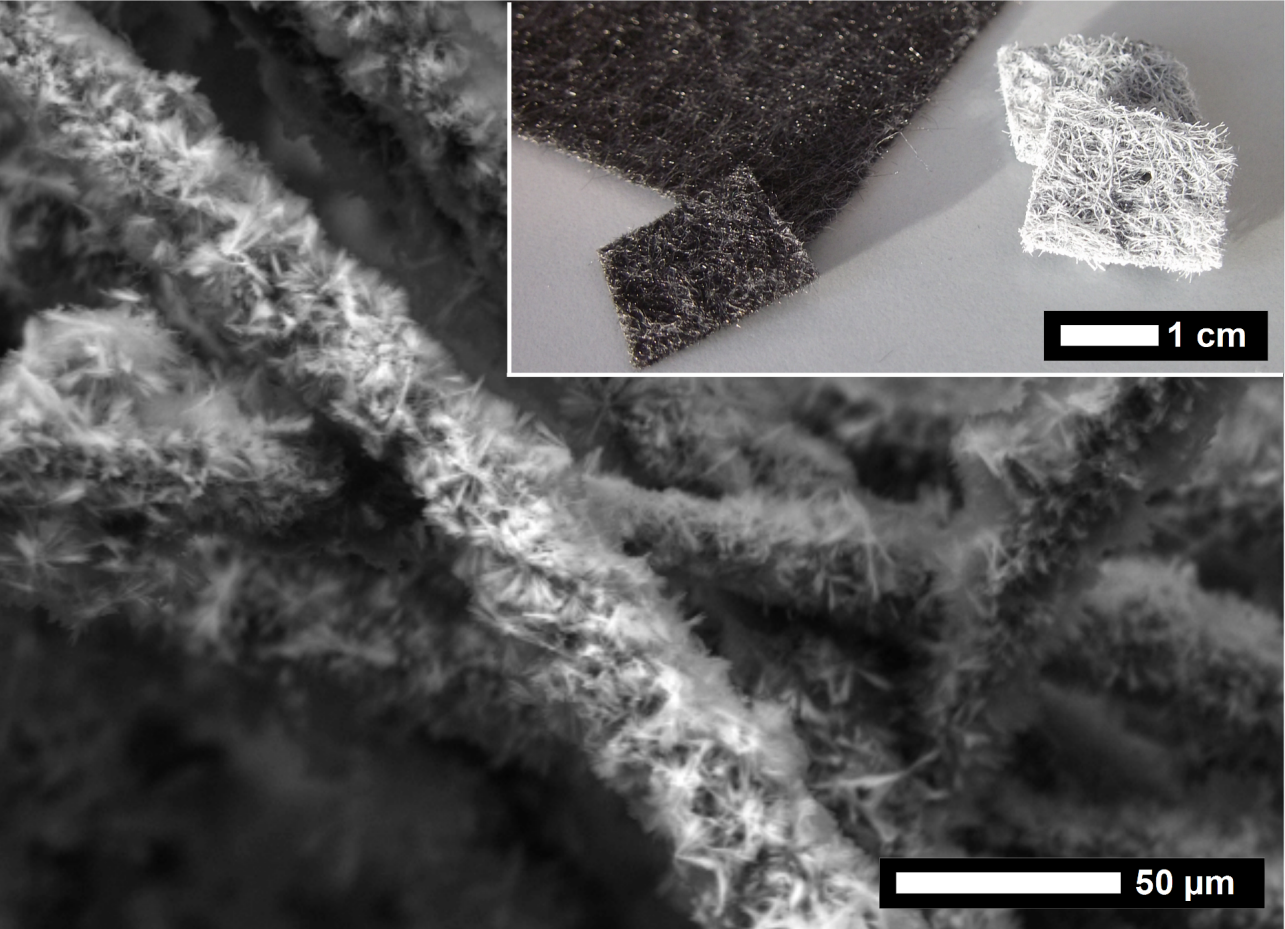
Scanning electron micrograph of a carbon felt with a LPEI mediated silica coating. Inset: photograph of activated carbon felt as received compared with the silica coated felt.

An immobilization of LPEI aggregates is not only possible on hydrophilic surfaces of chopped carbon fibers, but can also be realized for contiguous forms of carbon fibers. The latter is favorable for applications in the field of adsorption and for catalytic issues. It has to be mentioned that for a silica coating procedure of larger amounts of chopped carbon fibers, which have a length of 3 mm, the dilution is a crucial point. The mutual hindrance in more concentrated dispersions leads to a non-exhaustive coating of the surface. For a more or less complete surface coating, the fiber density in the dispersion should not exceed 1.5 g/L. This challenge and a potential interlocking of silicified fibers are insignificant for a coating process of carbon fiber felts. An activated carbon felt, which is intended for adsorption issues, was used as a commercially available test specimen for a LPEI mediated silica coating procedure. The felt can be immersed in the LPEI and TMOS solution and simply separated from the polyamine and the precursor solution resulting in an exhaustive surface coating (Figure 10, Supporting Information File 1, Figure S9). The fiber diameter increases from 17 µm, for the neat fibers, to about 35 µm, for the composite fibers including the nanostructured silica shell. Thermogravimetric analysis of the silica coated activated carbon felt shows that the silica content of the composite fiber is with a value of 51 wt % only slightly increased in comparison to the chopped carbon fibers.

The flexibility of the neat carbon fiber felt is preserved during mineralization with the result that the coated carbon fiber felt can be bended a few times. However, progressive bending is associated with a loss of silica and cracks in the silica shell. For very thick silica coatings, which can be realized for instance by calcium addition (Figure 9b), the hybrid fiber is more rigid and cracks in the shell will occur faster.

**Figure 11:**
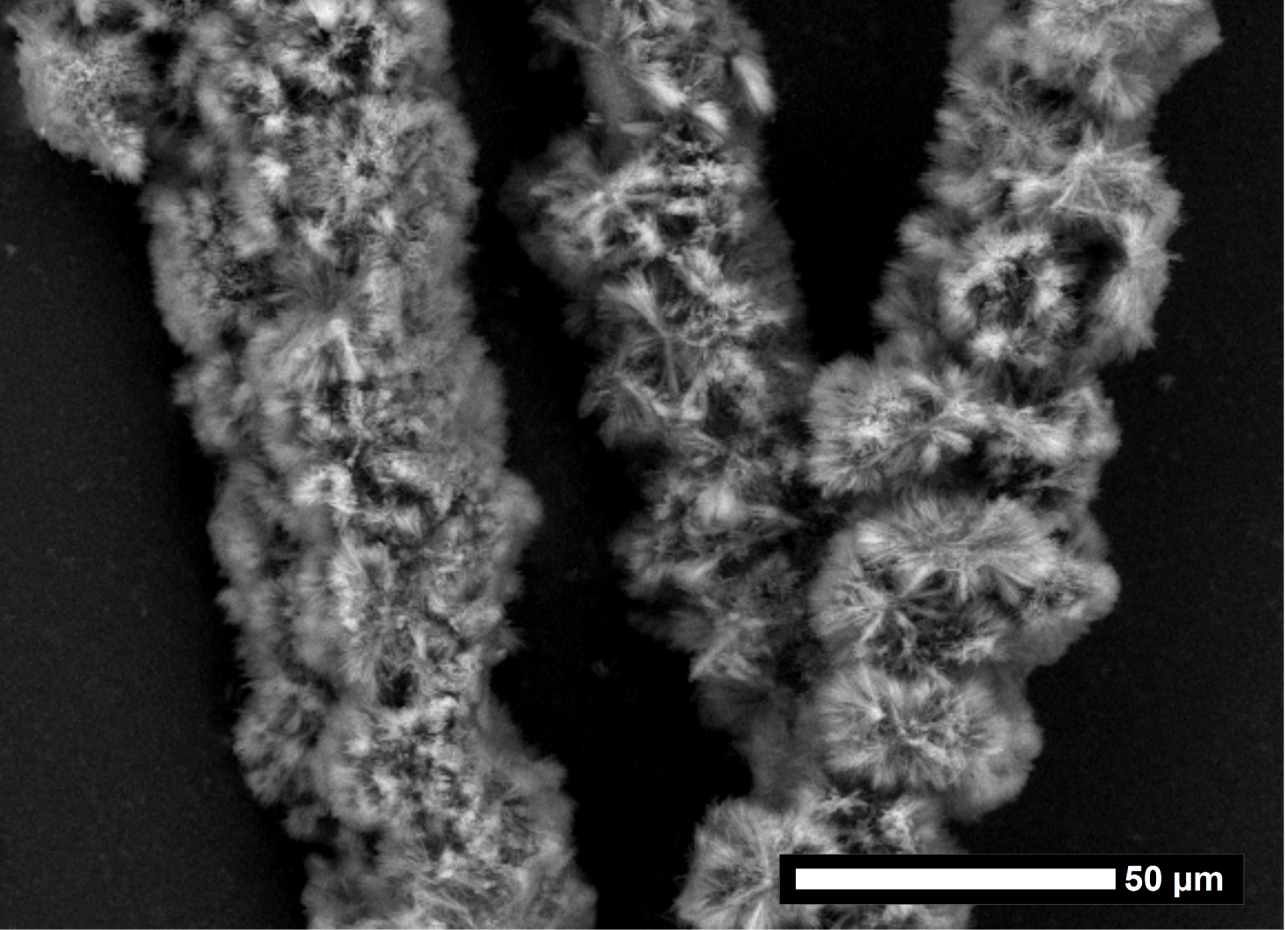
Scanning electron micrograph of silicon carbide fibers coated with a silica shell mediated by LPEI.

The facile transferability of the presented coating procedure and the adaptability to other fiber materials are illustrated by using silicon carbide fibers (Figure 11). Again, the molecular-level interactions of the alternative fiber surface and LPEI are important for facilitation of the polyamine self-assembly. Polyethylene based fibers are a less attractive substrate. However, an exhaustive coating can be easily achieved for fibers consisting of silicon carbide, aramid, steel, glass and basalt (Supporting Information File 1, Figure S10).

## Conclusion

A site-selective biomimetic silica deposition was accomplished via immobilization of linear polyamines on the surface of carbon fibers and a subsequent silicification on the basis of sol–gel chemistry. In the first approach, a covalent linkage of the short-chain polyamine tetraethylenepentamine to carbon fibers via amide functional groups was achieved. Owing to the small size of the TEPA molecule, the silica shell is confined to the nanometer scale.

LPEI was used in the second approach, i.e., a self-organizing of LPEI on the carbon fiber surface in the form of crystalline aggregates. The formation of the hierarchically structured silica shell strongly depends on the LPEI aggregates formed from the LPEI aqueous solution. The nanostructured silica shell is a unique characteristic of the presented method and can be easily controlled by altering synthesis conditions. In all cases, the main building unit consists of nanotubes with a LPEI core, a silica shell and a diameter between 10 and 30 nm. These primary building units are arranged to more complex and hierarchical structures depending on a wide range of factors. For the standard conditions, the nanotubes form a silica shell around the carbon fiber with a spatial extent of about 9 µm.

The presented coating procedure using LPEI is suitable for the deposition of thick silica coatings onto carbon fibers in the micrometer range. In addition, the silica shell exhibits a fine-structure in the nanometer regime. Both features are not feasible by means of conventional coating procedures such as chemical vapor deposition. The hierarchically and nanostructured silica shell introduces a high external surface area and, after removal of the LPEI template, a high internal surface area as well. It might be possible to enable access to new fields of application for carbon fibers in their contiguous form for catalytic applications and for adsorption purposes.

## Experimental

### Preparation

**Synthesis of linear poly(ethylenimine).** LPEI was synthesized by hydrolysis of the precursor polymer poly(2-ethyl-2-oxazoline) (PEOX, Sigma-Aldrich, average *M*_w_ = 50,000 g/mol corresponding to an average polymerization degree of ≈500, PDI ≈ 3–4), in a similar way as described elsewhere (Supporting Information File 1, Figure S11) [14]. Briefly, hydrolysis was achieved under refluxing at 100 °C in 5 M HCl aqueous solution of PEOX for 15 h with a molar ratio of HCl to acetylethylenimine monomer unit of 3:1. The obtained precipitate of the hydrochloride of LPEI was filtered off, washed with methanol, dried under ambient conditions and subsequently dissolved in water by neutralizing with 25 wt % aqueous ammonia solution. The clear polymer solution was transferred into dialysis tubing (Aldrich, cellulose membrane, typical molecular weight cut-off: 3500 kDa) and the dialysis was carried out against 5 M ammonia solution for 3 days with 6 changes of the ammonia solution. The obtained white crystalline LPEI was washed with water and acetone and dried in a desiccator over silica gel under vacuum. The remaining water content is about 20 wt % as determined by thermogravimetric analysis.

**Modification of carbon fiber surface by nitric acid oxidation followed by reaction with tetraethylenepentamine and subsequent silicification.** For chemical modification, chopped carbon fibers with a water-soluble sizing were used (Tenax HT-A C124, Toho Tenax, average length: 3 mm) and the sizing was removed by washing with deionized water several times. To increase the number of surface-exposed carboxylic acid groups, the dried fibers (10 g) were oxidized in 65% nitric acid (200 mL) at 115 °C for 90 min. The fibers were washed repeatedly with deionized water prior to drying at 60 °C. Tetraethylenepentamine was grafted to the carbon fiber surface by heating 1 g fibers in an excess of TEPA reagent (10 mL) at 180 °C for 6 h. The fibers were filtered off, washed with water and methanol and dried at 60 °C. These modified fibers were silicified in a mixture of ethanol, water and TMOS (for each 33 vol %) over a period of 40 min.

**Preparation of separate LPEI/silica hybrid particles.** Non-immobilized LPEI aggregates were obtained by heating 1 mL of an aqueous suspension of LPEI (1.25 wt %) to a temperature of ≈80 °C by immersing the glass tube in a thermostated water bath. Cooling down of the hot aqueous solution to room temperature resulted in a hydrogel or polymer particles, respectively, which were added to 1 mL of a mixture of tetramethyl orthosilicate (Aldrich) and ethanol (1:1 by volume) after a period of 12 h. The mineralization time was 40 min. The precipitated silica particles were separated via centrifugation, washed with water and ethanol and dried at ambient conditions leading to white silica powders. Calcination of the LPEI/silica hybrid material was performed in air atmosphere with a heating rate of 1.5 K/min up to a maximum temperature of 475 °C at which the sample was kept for 90 min. In the case of metal-ions containing LPEI aggregates, the water phase of the aqueous LPEI suspension was substituted by a copper nitrate solution and a calcium nitrate solution, respectively. The ratio between the metal ion and the ethylenimine monomer unit was set to 1:30.

**Modification of carbon fiber surfaces by LPEI self-assembly and subsequent silicification.** The sizing of chopped carbon fibers (Tenax HT C261, Toho Tenax, sizing with an epoxy compatible polymer) with an average length of 3 mm and a diameter of 7 µm was removed by calcination in a furnace in air atmosphere (heating rate: 200 °C/h, maximum temperature: 425 °C, isothermal time: 5 h). The preparation procedure for the LPEI mediated silica coatings on carbon fibers was similar to the one used for the preparation of LPEI/silica composite particles including now the direct addition of carbon fibers (20 mg/mL) to the LPEI suspension prior to heating the suspension to 80 °C. The hot solution was cooled down to room temperature, the fibers were filtered off after 12 h by means of a sieve (*d* = 0.56 mm) and silicified in a solution of 3 vol % TMOS in ethanol/water (1:1 by volume). The fibers were washed with water and ethanol and dried at 60 °C.

Other fibers used for the LPEI mediated silica coating process were anodic oxidized carbon fibers, silicon carbide fibers and an activated carbon felt. The high-tenacity carbon fiber was supplied by SGL Carbon in the process state after anodic oxidation without further sizing (Sigrafil C30 T050 us). Silicon carbide fibers (Goodfellow GmbH; average diameter 15 µm) were used as received. The activated carbon felt was kindly supplied by Unitika Ltd. and was pretreated by heating for 5 h at 190 °C in a thermostatically controlled oven in air atmosphere.

### Characterizations

Thermogravimetric analysis (TGA) was performed with a TA Instruments Q500 Thermogravimetric Analyzer. Nitrogen gas sorption isotherms of LPEI/silica composite particles at 77 K were recorded with a Quantachrome NOVA 2000 Series instrument. Prior to measurements, the samples were heated for 2 h in vacuum at 110 °C (LPEI/silica hybrid particles) and 150 °C (calcined silica nanotubes), respectively. Energy-dispersive X-ray spectroscopy (EDX) was performed with a XL30 FEG ESEM (environmental scanning electron microscope, FEI/Philips) equipped with an EDAX SiLi detector. The ESEM was used for electron micrographs in low-vacuum mode (0.6 mbar) and a Leo 1530 Gemini SEM (Zeiss) was used for recording scanning electron micrographs in high-vacuum mode, in all cases without sputtering the samples. TEM investigations were performed using a JEOL 2100F microscope with a FEG electron source operated at 200 kV. The microscope is equipped with a Gatan image filter. Holey carbon-coated copper grids were used for sample preparation. Atomic force topographic images were sampled by means of an Agilent 5500 AFM with MAC III controller operating in tapping mode. A polynomial background subtraction was applied for image processing.

## Supporting Information

File 1 Experimental details of acid catalyzed silica deposition, TGA charts and scanning electron micrographs.
Supporting information features experiments for silica deposition under acid catalyzed conditions to highlight the importance of polyamine localization on the carbon fiber surface for site selective mineralization. In addition, TGA profiles of both the LPEI/silica hybrid particles and carbon fibers covered by silica nanotubes are given. Further scanning electron micrographs are demonstrating the convenient control over the nanostructured silica shell and the transferability of the introduced synthesis method to versatile fiber substrates.
