# Supplementary material for: Preparation of thick silica coatings on carbon fibers with fine-structured silica nanotubes induced by a self-assembly process
Source: Beilstein J Nanotechnol. 2017 May 26;8:1145–55. doi: 10.3762/bjnano.8.116 (PMC5480351; doi:10.3762/bjnano.8.116)
Supplement: File 1 — Experimental details of acid catalyzed silica deposition, TGA charts and scanning electron micrographs. Supporting information features experiments for silica deposition under acid catalyzed conditions to highlight the importance of polyamine localization on the carbon fiber surface for site selective mineralization. In addition, TGA profiles of both the LPEI/silica hybrid particles and carbon fibers covered by silica nanotubes are given. Further scanning electron micrographs are demonstrating the convenient control over the nanostructured silica shell and the transferability of the introduced synthesis method to versatile fiber substrates. [file Beilstein_J_Nanotechnol-08-1145-s001.pdf]

Supporting Information

for

Preparation of thick silica coatings on carbon fibers with fine-structured silica nanotubes induced by a self-assembly process

Benjamin Baumgärtner[1], Hendrik Möller[2], Thomas Neumann[3] and Dirk Volkmer[*1]

Address: [1]Chair of Solid State and Materials Chemistry, University of Augsburg, Augsburg 86159, Germany, [2]Schwenk Zement KG, Ulm 89077, Germany and [3]Schwenk Zement KG, Karlstadt 97753, Germany

Email: Dirk Volkmer - dirk.volkmer@physik.uni-augsburg.de

* Corresponding author

Experimental details of acid catalyzed silica deposition, TGA charts and scanning electron micrographs

Acid catalyzed sol-gel process in the presence of unmodified carbon fibers

Experimental: The sizing of chopped carbon fibers (Tenax HT C261, Toho Tenax) with an average length of 3 mm and a diameter of 7 µm was removed by calcination in a furnace in air atmosphere (heating rate: 200 °C/h, maximum temperature: 425 °C, isothermal time: 5 h). These carbon fibers, i.e. without an additional polyamine coating, were subjected to an acidic silica precursor solution. Prolonged reaction times of 30 h were realized for diluted sodium silicate solution (~ 3.4 wt% SiO_2, ~ 1.0 wt% Na_2O, VWR Chemicals) at pH 2 (adjusted with hydrochloric acid) and ambient conditions. The carbon fibers were filtered off, washed with water and ethanol and dried at ambient conditions.

Results & Discussion: Acid-catalyzed silica deposition onto carbon fibers via a sol-gel process without a polyamine mediation was experimentally realized by employing less reactive but more economically priced sodium silicate solution. This silica precursor was diluted with water, the pH adjusted to a value of 2 (the reactivity in the acidic range reaches a minimum around this pH value) and the carbon fibers were kept for 30 h in the silica precursor solution. The carbon fibers were separated and analyzed by means of EDX and AFM. Energy-dispersive X-ray spectroscopic measurements were performed by focusing the electron beam of the electron microscope on several different sections of the carbon fiber surface. Presence of silicon was detected and a coating further confirmed by atomic force microscopy in the form of distinct granular structures on the fiber surface (Figure S1). Thermogravimetric analysis is less informative due to secondary, i.e. not on the fiber surface localized, silica particles, which emerged from the continuous phase and can reach dimensions up to several hundred micrometers. The presence of these polydisperse silica particles can be visualized by electron micrographs (Figure S2) illustrating that the silica deposition is not limited to the carbon fiber surface in the acid catalyzed way of sol-gel chemistry.

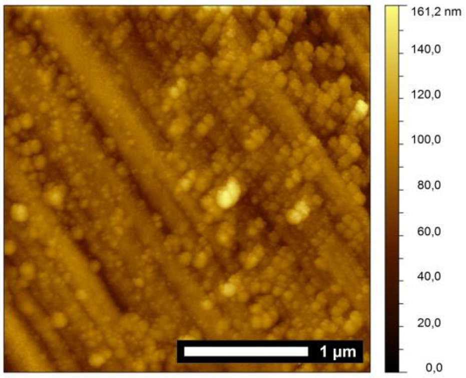

Figure S1: Atomic force micrograph of carbon fibers after 30 h of treatment in acidic sodium silicate solution.

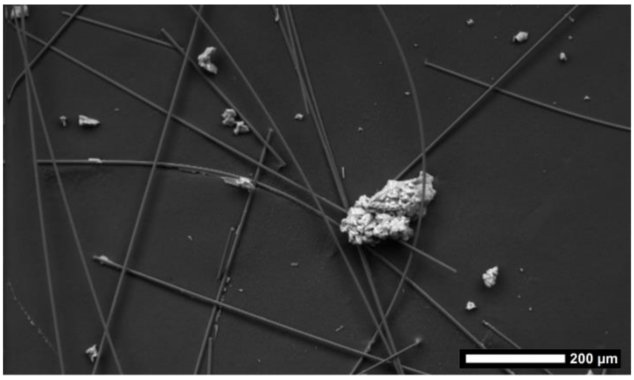

Figure S2: Scanning electron micrograph of carbon fibers after 30 h of treatment in acidic sodium silicate solution.

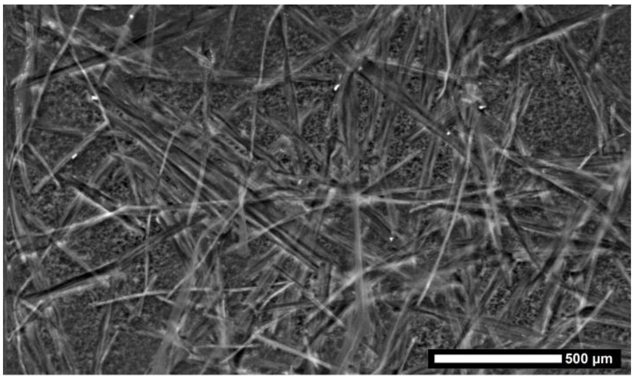

Figure S3: Scanning electron micrograph of the TGA residue of TEPA-modified and silicified carbon fibers.

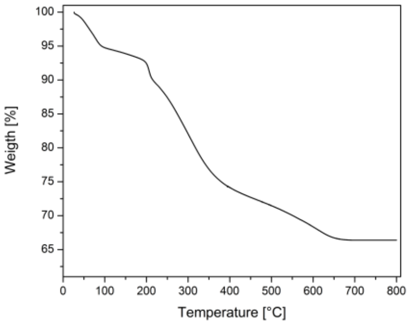

Figure S4: Thermogravimetric analysis of LPEI-silica hybrid particles (heating rate: 5 K/min; air atmosphere).

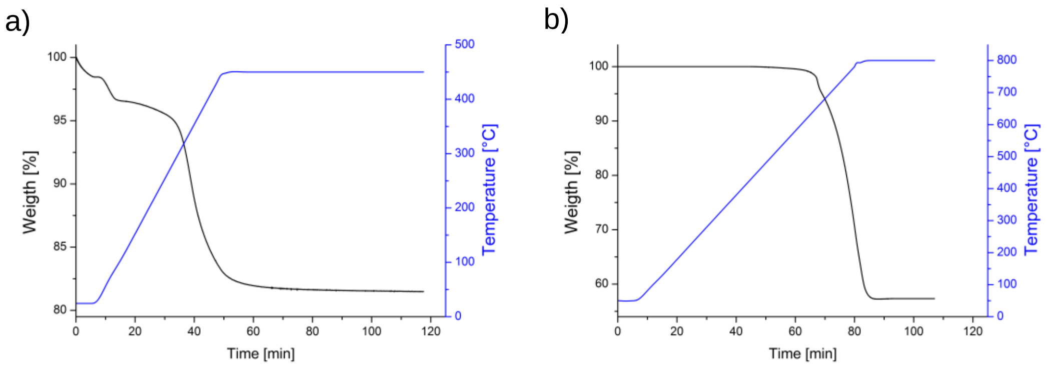

Figure S5: Thermogravimetric analysis of carbon fibers with a silica shell mediated by LPEI. a) First heating cycle in nitogen atmosphere (heating rate: 10 K/min; max. temp. 450 °C for 60 min.) causes a mass loss due to LPEI decomposition and water evaporation. b) Second heating cycle of the same sample in air atmosphere (heating rate: 10 K/min; max. temp. 800 °C for 20 min.)), leading to oxidative decomposition of the carbon fiber core.

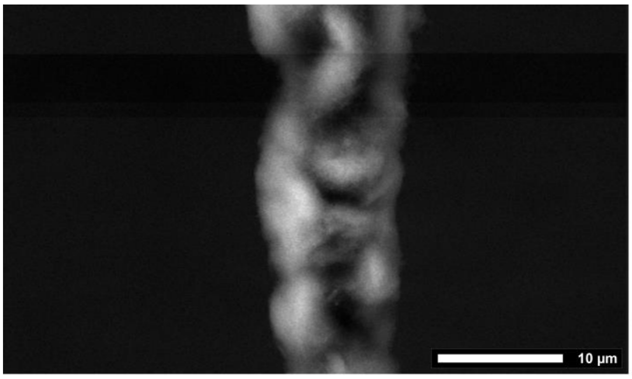

Figure S6: Scanning electron micrograph of a LPEI mediated silica shell on a carbon fiber obtained from a faster cooling process during LPEI self-assembly.

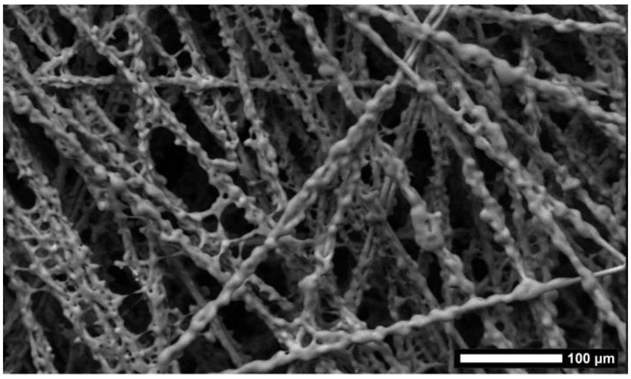

Figure S7: Scanning electron micrograph of LPEI mediated silica shells on carbon fibers followed by sintering at 1100 °C in argon atmosphere.

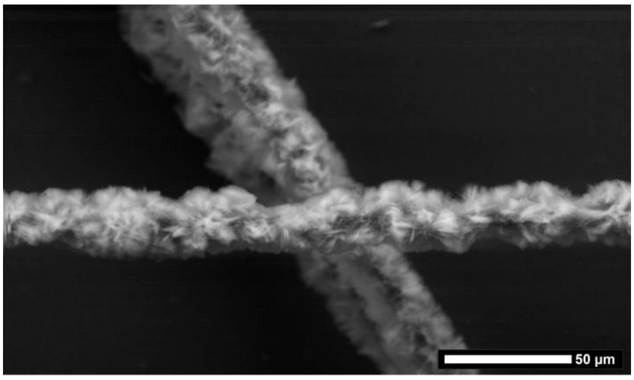

Figure S8: Scanning electron micrograph of LPEI mediated silica shells grown on sulfonated carbon fibers. For sulfonation the water soluble sizing was removed by washing, 2.5 g chopped carbon fibers were suspended in 100 mL benzene and refluxed at 81 °C for 2 h with addition of 5.0 g of dibenzoyl peroxide. The fibers were washed with chloroform, dried and heated at 80 °C for 2 h in fuming sulfuric acid (60% SO_3).

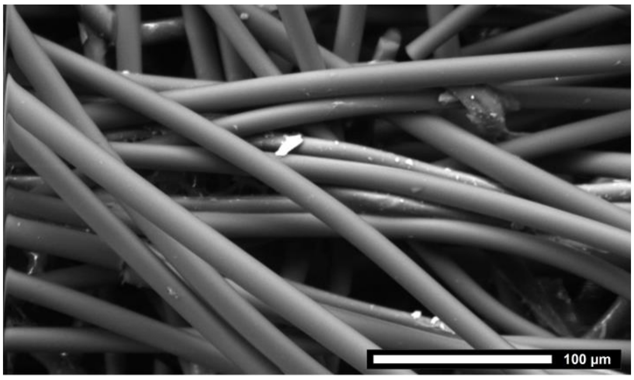

Figure S9: Scanning electron micrograph of the activated carbon felt as received (fiber diameter: 17 µm).

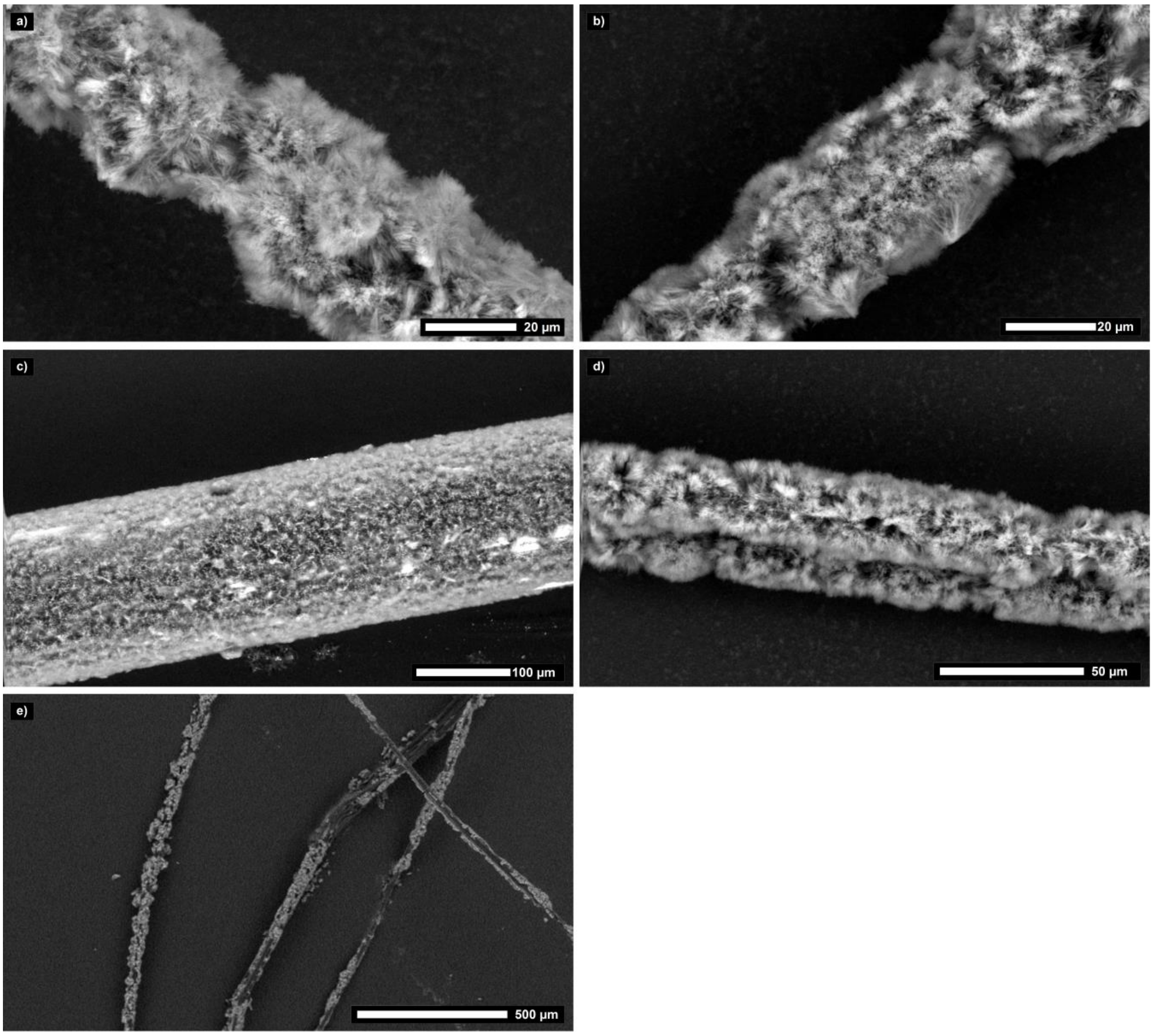

Figure S10: Scanning electron micrographs of different fiber substrates coated with a silica shell mediated by LPEI. a) Basalt, b) aramid, c) steel, d) glass and e) Dyneema (polyethylene based).

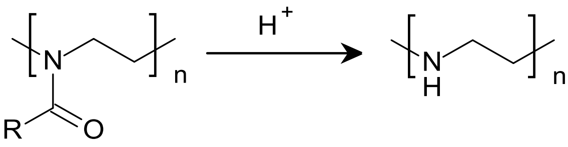

Figure S11: Schematic illustration of linear poly(ethyleneimine) synthesis through acidic hydrolysis of pol(2-alkyl-2-oxazoline).
